# FlexFlux: combining metabolic flux and regulatory network analyses

**DOI:** 10.1186/s12918-015-0238-z

**Published:** 2015-12-15

**Authors:** Lucas Marmiesse, Rémi Peyraud, Ludovic Cottret

**Affiliations:** INRA, Laboratoire des Interactions Plantes-Microrganismes (LIPM), UMR441, 24 chemin de Borde Rouge - Auzeville, CS52627, Castanet-Tolosan Cedex, F31326 France; CNRS, Laboratoire des Interactions Plantes-Microrganismes (LIPM), UMR2594, 24 chemin de Borde Rouge - Auzeville, CS52627, Castanet-Tolosan Cedex, F31326 France

**Keywords:** Metabolism, Regulatory network, Flux balance analysis, Genome-scale models, Logical models, Steady-state, Multi-state, Java

## Abstract

**Background:**

Expression of cell phenotypes highly depends on metabolism that supplies matter and energy. To achieve proper utilisation of the different metabolic pathways, metabolism is tightly regulated by a complex regulatory network composed of diverse biological entities (genes, transcripts, proteins, signalling molecules…). The integrated analysis of both regulatory and metabolic networks appears very insightful but is not straightforward because of the distinct characteristics of both networks. The classical method used for metabolic flux analysis is Flux Balance Analysis (FBA), which is constraint-based and relies on the assumption of steady-state metabolite concentrations throughout the network. Regarding regulatory networks, a broad spectrum of methods are dedicated to their analysis although logical modelling remains the major method to take charge of large-scale networks.

**Results:**

We present FlexFlux, an application implementing a new way to combine the analysis of both metabolic and regulatory networks, based on simulations that do not require kinetic parameters and can be applied to genome-scale networks. FlexFlux is based on seeking regulatory network steady-states by performing synchronous updates of multi-state qualitative initial values. FlexFlux is then able to use the calculated steady-state values as constraints for metabolic flux analyses using FBA. As input, FlexFlux uses the standards Systems Biology Markup Language (SBML) and SBML Qualitative Models Package (“qual”) extension (SBML-qual) file formats and provides a set of FBA based functions.

**Conclusions:**

FlexFlux is an open-source java software with executables and full documentation available online at http://lipm-bioinfo.toulouse.inra.fr/flexflux/. It can be defined as a research tool that enables a better understanding of both regulatory and metabolic networks based on steady-state simulations. FlexFlux integrates well in the flux analysis ecosystem thanks to the support of standard file formats and can thus be used as a complementary tool to existing software featuring other types of analyses.

**Electronic supplementary material:**

The online version of this article (doi:10.1186/s12918-015-0238-z) contains supplementary material, which is available to authorized users.

## Background

Analysis of metabolic networks is extensively used as a direct reflect of the phenotype of living cells. Moreover, the increasing amount of available omics data has encouraged the reconstruction of a significant number of genome-scale metabolic networks [1]. A very popular approach to study metabolic networks is the use of a constraint-based method called Flux Balance Analysis (FBA) [2]. FBA is based on the steady-state assumption which results in mass balance through the metabolic network. Given a biological objective (like growth or metabolite production), the space of optimal solutions for the reaction fluxes can be very quickly solved by linear-programming computation even for large networks. This calculation only relies on knowing reaction stoichiometry and user-defined input flux values. Different FBA-based analyses are implemented in a number of available software including COBRA Toolbox [3], COBRApy [4], CellNetAnalyser [5], SurreyFBA [6], OptFlux [7], FASIMU [8] and SBRT [9]. For a review of differences and specificities of some of these tools, see [10].

However, metabolic network analysis alone cannot explain the differences observed between two differentiated cells of an organism, or the behaviour of a versatile micro-organism given a particular environmental condition. Indeed, cells have evolved regulatory networks to integrate environmental signals or acquired differentiated states that result in modulation of gene expression. Therefore this specific gene expression triggers specific phenotypes depending on the environmental constraints or cell differentiation. Thus, computing embedded metabolic and regulatory networks is a paramount objective in order to study complex cell phenotypes. For instance, Buescher et al., by integrating metabolic and regulatory network analyses, simulated different regulation strategies for controlling nutritional shifts and compared their evolutionary benefit. They thus succeeded to identify the key regulatory events involved in the metabolic adaptative response to nutritional transitions in *Bacillus subtilis* [11].

Many methods are available in the literature for the analysis of regulatory networks, going from the most elaborated (based on differential equations) to the simplest (Boolean models). Some of these methods are reviewed here [12–14]. For large networks, qualitative multi-state models seem to be a good compromise between the number of required parameters and the quality of simulations [15]. In these types of models, the components display a finite number of possible states, and their values are updated via logical rules composed of the states of other components. This makes the search for steady-states easier than with continuous models. They have the advantage of not requiring any kinetic parameter for simulations, like FBA, and they provide more modelling precision than Boolean models. A growing number of qualitative networks is available to the community [16] through platforms like The Cell Collective [17]. This sharing of qualitative models is facilitated by the development of SBML-qual [18], a standard XML (Extended Markup Language) based format designed to represent multi-state qualitative models based on the SBML format [19]. Some software tools have integrated this format and can perform qualitative network analyses : The Cell Collective [17], CellNetAnalyser [5], GINsim [20], CellNOpt [21] and BoolNet [22].

Different methods have been developed to connect metabolic network and regulatory network analyses. Most of these methods are dynamic: regulatory FBA (rFBA) [23], Probabilistic Regulation Of Metabolism (PROM) [24], iFBA [25]. This allows to take into account a feedback of FBA on the regulatory network by considering metabolite concentrations. However it requires many FBA optimisations and differential equations to update concentrations. In rFBA, regulatory rules can constrain a reaction only by setting its flux value to 0. PROM [24] is based on regulatory network reconstruction through inference from microarray data and is able to constrain a reaction to a certain percentage of its maximal flux value. This percentage corresponds to the estimated probability of activation of the gene associated to the reaction. This method requires data from many microarray experiments (several hundreds). In the iFBA method [25], the authors integrated a set of ordinary differential equations (ODEs) to rFBA. This allows to accurately predict phenotype of diauxic growth of *Escherichia coli* but requires kinetic parameters for the ODEs. Another method called steady-state rFBA (SR-FBA) [26] is not based on a dynamic simulation but on steady-state. It includes the Boolean rules in the optimisation process of the FBA using mixed integer linear programming (MILP), and thus finds a steady-state for both metabolic and regulatory networks. However, this method does not allow to model feedback loops.

In all of these methods, regulatory rules can only constrain reactions to one single flux value.

In this context, we have developed FlexFlux, a tool that allows the analysis of both qualitative regulatory networks and genome-scale metabolic networks. FlexFlux is the first metabolic flux analysis tool that natively integrates regulatory networks in all of its functions. Regulatory and metabolic networks can be analysed either separately or together. When analysed together, regulatory network states are used to constrain the FBA. The regulatory network is considered as known in FlexFlux and must be provided by the user.

The key features of FlexFlux are the following:

- FlexFlux supports qualitative multi-state regulatory networks, including the simplest variant: Boolean networks. The regulatory networks can be composed of various types of biological components. The multi-state nature of the network allows for instance to simulate different levels of gene expression, which is not possible with a Boolean model.
- FlexFlux allows a translation of the discrete qualitative states of the regulatory networks into user-defined continuous intervals. This permits to constrain a reaction flux with different intervals according to a qualitative state and not to a unique value like in the methods presented above. This translation is also used for the input (initial values) of the regulatory network to obtain qualitative states from metabolite external concentrations.
- To constrain metabolic fluxes with a regulatory network, FlexFlux performs a regulatory steady-state analysis (RSA). See below for a description of the algorithm implemented in FlexFlux to perform the RSA.
- It supports the SBML-qual file format for the description of the regulatory network. FlexFlux is the first flux analysis software to support this file format.

These features are explained in more details in the next sections.

## Implementation

FlexFlux is an open source java software distributed for Windows and Linux. It can be used in command line or with a graphical interface.

### General architecture

**Fig. 1:**
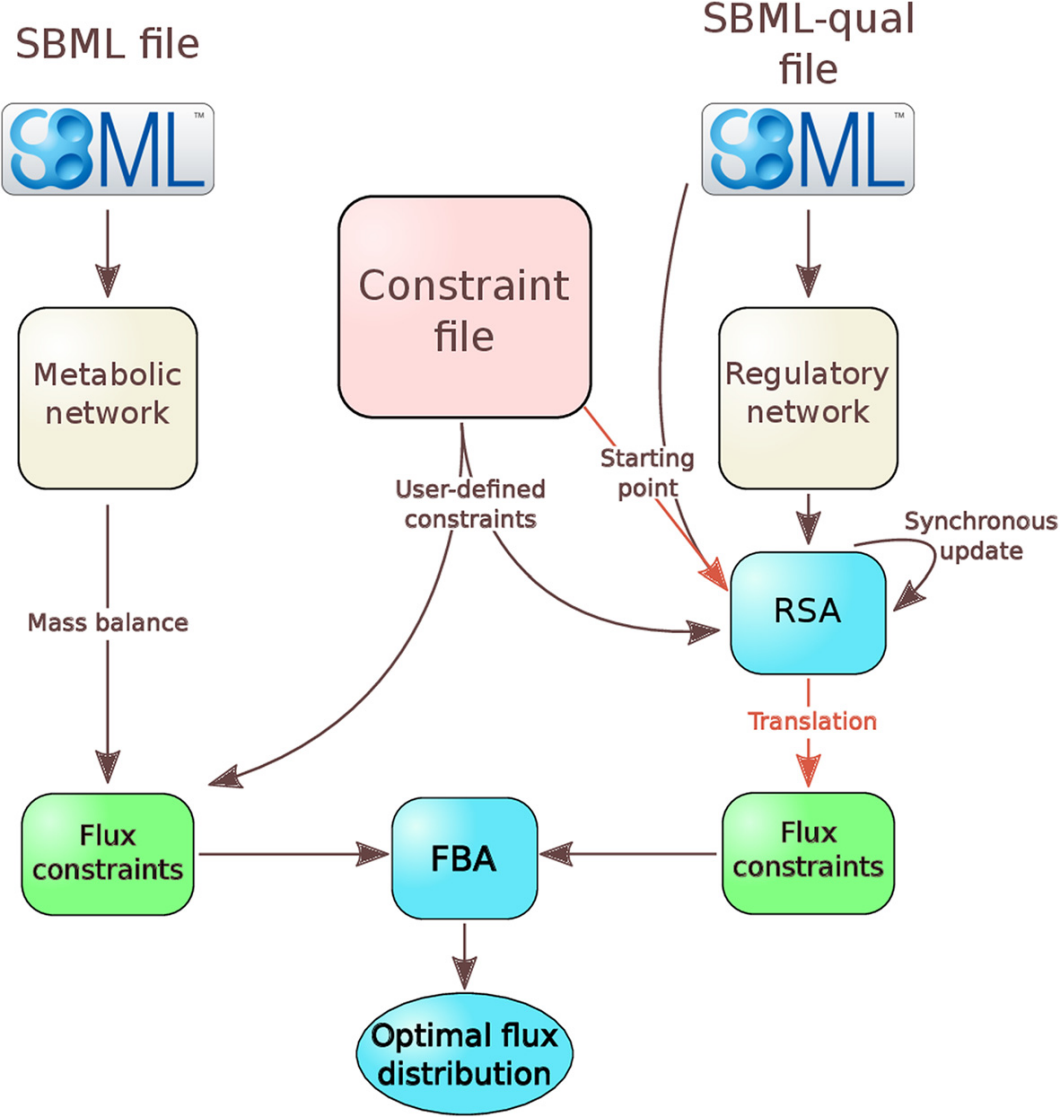
General architecture of FlexFlux. FlexFlux takes as an input two SBML files and a constraint file : one SBML file for the description of the metabolic network, and the Qualitative extension for the regulatory network. The constraint file specifies the objective function and can contain constraints. From the metabolic network definition, FlexFlux creates mass balance constraints that will be used for the flux balance analysis (FBA). The regulatory network is analysed by a regulatory steady-state analysis (RSA). The starting point of the RSA comes from both SBML-qual and constraint files. Then the qualitative states are translated into constraints for the FBA. FBA and RSA can also be run independently
- A SBML-qual file which describes a qualitative regulatory network : components, initial values and update rules.
- A constraint file, which contains the objective function for the FBA and may contain additional constraints defined by the user. Note that initial values of the regulatory network components can be specified in this file. If so, they overwrite the ones present in the SBML-qual file.

Depending on the function, FlexFlux can take as input three files (Fig. 1):

- A SBML file which describes the metabolic network.

FlexFlux is able to perform both regulatory steady-state analysis (RSA) and metabolic network analyses using FBA. It can connect both analyses by constraining FBA with steady-states obtained with the RSA (Fig. 1).

### Regulatory network analysis

To analyse regulatory networks in FlexFlux, we designed and implemented an algorithm called Regulatory Steady-state Analysis (RSA). Its goal is to obtain a single “steady-state” constraint for each component of the network from initial values. A constraint is defined by a lower and an upper bound defining a range of possible values of the component. To facilitate connection with quantitative methods like FBA, FlexFlux supports quantitative inputs and outputs by translating them into qualitative states.

**Fig. 2:**
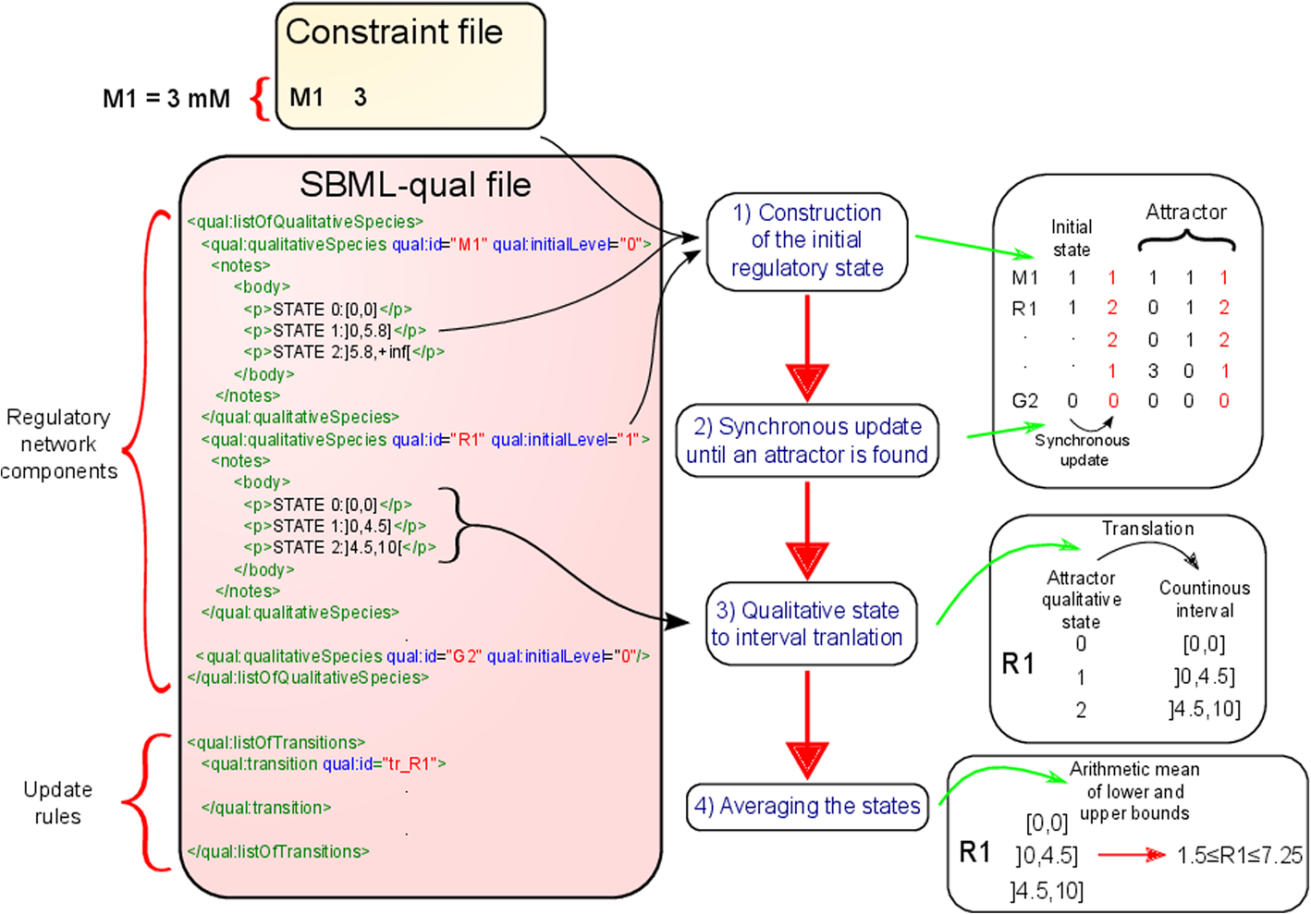
Regulatory network steady-state analysis of FlexFlux. 1) The initial state is constructed from qualitative values present in the SBML-qual file (for R1) and quantitative values in the constraint file that are translated into qualitative values by the equivalences written in the SBML-qual network (for M1). 2) From this initial state, the network is iteratively updated using a synchronous update of all components. Integer values represent qualitative states of the components of the network. The update of the network state stops when a state which was already calculated is found (the states shown in red here). The attractor is composed of this state plus the states between the two identical states. 3) The link between qualitative states and continuous intervals must be specified in the SBML-qual file describing the regulatory network in the notes of the *QualitativeSpecies* tag. For each component where they are provided, all the states of the attractor are translated into intervals. The example is shown for R1 here. 4) When the attractor contains more than one state (cyclic attractor), the average of the upper bounds and lower bounds of all the intervals is used to form a steady-state constraint for components containing equivalences

The SBML-qual file provided to FlexFlux must respect the specifications described in [18]: it must contain a list of *QualitativeSpecies* with a specified initial level, and a list of *Transitions* corresponding to the logical rules for the update of a species (Fig. 2). The *QualitativeSpecies* will correspond to the components of the regulatory network. In order to use FlexFlux’s translation, the equivalences between qualitative states and continuous intervals must be specified as notes in the *QualitativeSpecies* tag (Fig. 2). This information will be used during the regulatory steady-state analysis algorithm. Note that specifying this equivalence is not mandatory if the species does not use quantitative inputs or outputs. The file is read with the JSBML library [27].

The algorithm consists in four successive steps (Fig. 2):

- **Construction of the initial regulatory network state**. The SBML-qual file must contain initial qualitative values for each component of the regulatory network. However, they can be overwritten by the constraint file. If the constraint file contains an initial value for a component, it is translated into a qualitative state, and used as initial value for the regulatory network (Fig. 2). This allows simulating different external metabolite concentrations.
- **Search for an attractor**. An attractor can be defined as a set of network states toward which the network evolves. To find an attractor, the network state is updated from the initial values via a synchronous update of all the components according to their corresponding *Transition* (Fig. 2). The update being synchronous, a state is defined by the previous one, so that once a state already found is encountered, no new state can be reached and an attractor has thus been found. An attractor of size one is called a point attractor, whereas an attractor of size higher than one is called a cyclic attractor. In the latter case, it corresponds to all the states between the two identical states plus one of the two identical states (Fig. 2). A cyclic attractor can be seen as a loop that the network states will infinitely go through.
- **Translation of qualitative states into intervals**. In order to define the quantitative output of the regulatory steady-state analysis for the components harbouring the equivalences described before, the values of the states contained in the attractor are translated into corresponding continuous intervals (Fig. 2). If these equivalences (qualitative to continuous intervals) are not specified for a component, the output for this component remains qualitative.
- **Averaging the states in the case of a cyclic attractor**. This step is performed for cyclic attractors (attractors that have more than one state). From a cyclic attractor, a single steady-state constraint is defined for each species. If quantitative equivalences have been specified, the final bounds of the constraint are determined by calculating the arithmetic mean of the bounds corresponding to each state found in the attractor. In the case of species without quantitative equivalences, the constraint corresponds to a single value equal to the arithmetic mean of the component states found in the attractor.

### Constraining FBA with regulatory steady-state constraints

Three types of biological components of the regulatory network can have an effect on the FBA: reactions, genes and external metabolites.

The steady-state constraints obtained for reactions from RSA are directly added to the FBA model constraints.

The link between a gene value obtained from RSA and FBA is made through gene-protein-reaction (GPR) associations that can be specified in the SBML file describing the metabolic network [28]. In the case of a GPR association, if the regulatory steady-state constraint sets the gene to 0, the associated reaction flux is set to 0. If the value of the gene is different from 0, no constraint is added on the reaction flux.

In the metabolic model, external metabolites are imported in the model by exchange reactions. Constraints on these exchange reactions mimic different environmental conditions. In FlexFlux, if a null value is attached to an external metabolite, the uptake flux value for the corresponding exchange reaction will be constrained to 0.

### Metabolic network analysis

The metabolic network must be described in a SBML file. FlexFlux constructs the mass balance constraints required for the FBA, and reads the objective function and additional constraints from the constraint file. If any, the constraints obtained from the regulatory network steady-state analysis are added to the model.

The optimisation process is done by a linear programming solver. At present, FlexFlux is compatible with two solvers: ILOG CPLEX [29] and the GNU Linear Programming Kit (GLPK) [30].

### FlexFlux functions

At the moment, FlexFlux contains 13 functions. Because some functions require many successive FBA, FlexFlux implementation was also focused on computation speed. This is done by using parallelism and solver warm-starts capabilities [31].

Regulatory steady-state analysis (RSA) is integrated in every FlexFlux function, but can also be launched by itself. One function (Time-Dependent analysis) is not based on steady-state analysis of the regulatory network but on a dynamic analysis with iterative updates of the network. This allows to take into account a change in metabolite concentration resulting from FBA.

**Fig. 3:**
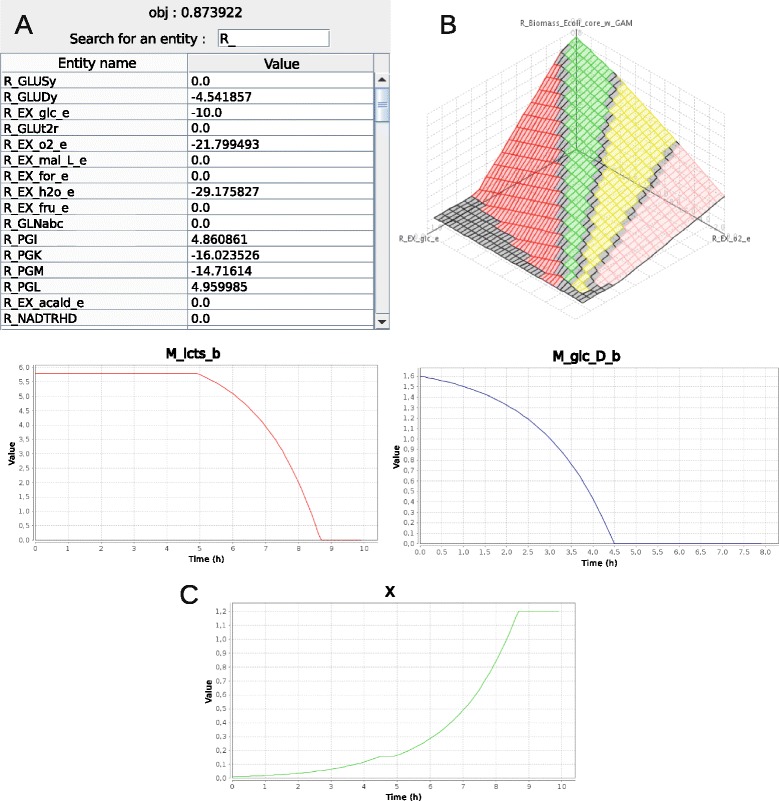
Screenshots of some of FlexFlux graphical results. **a** Result of a Flux Balance Analysis performed on a *Escherichia coli* metabolic network with a maximisation of the biomass. The value of the objective function is shown in the upper part. The value of each network component can be found in the table. **b** Result of a 3D phenotype phase analysis performed on *E. coli* metabolic network. The effect of the variation of both glucose and oxygen uptake fluxes on the biomass shows four distinct phenotype phases (shown here in four different colours). **c** Result of a time-dependent analysis performed on *E. coli* metabolic network with glucose and lactose available in the medium. It shows the evolution of the external concentration of glucose (M_glc_D_b), lactose (M_lcts_b) and the evolution of the cell density (X)

The detail of all functions inputs and outputs as well as more details, example files, command lines and graphical results are available on FlexFlux’s web site. Some of FlexFlux graphical outputs are shown in Fig. 3.

Some of these functions are briefly described below.

#### RSA and multi RSA

These two functions implement the algorithm of regulatory network analysis described above. Multi RSA allows performing a high number of RSA with multiple initial conditions on the same regulatory network. These multiple conditions can be randomly generated by another FlexFlux function called RandomConditions.

#### Flux balance analysis (FBA)

The basic function of FlexFlux. It finds the steady-states of the regulatory network if provided and uses it to add constraints for the FBA as previously described. The objective function used in the FBA is defined in the constraint file. A graphical result of a FBA is shown in Fig. 3 panel a.

#### Flux variability analysis (FVA)

The goal of this function is to compute the maximal and minimal values of all reaction fluxes, or a set of fluxes, when the objective function is optimized. To do that, FlexFlux performs a regulatory steady-state analysis, a FBA with the provided objective function. Then, the optimal value of the objective function is set as a constraint, and all reactions are consecutively minimised and maximised as objective functions of new FBA.

FlexFlux’s implementation of FVA uses the fastFVA approach [31]. When applied to a metabolic network of around 2000 reactions, it can be performed in less than 15 s on a computer with one processor (Intel ^*Ⓡ*^ Core™ i5-4590). A comparison with the computation time of FVA in other FBA software is detailed in Additional file 1 (Table S1).

#### Knockout analysis (KO)

Here the goal is to infer the effect of individual variables knockouts on the objective function. Thus, for each tested variable, a new constraint setting its value to 0 is added to the model. Then a RSA and a FBA are performed. Note that FlexFlux allows to perform a knockout in any component of the metabolic or the regulatory network (reactions, metabolites, genes, transcription factors, etc …).

#### Phenotypic phase analysis

This analysis permits to find specific metabolic network behaviours (phenotypic phases) [32] by varying one or two reaction fluxes, generating a 2D or a 3D graph respectively (Fig. 3 panel B). For increasing values of the provided reactions fluxes, FlexFlux optimises the objective function and calculates a shadow-price value for each point. In our case, the shadow-price value corresponds to how much the objective function value varies when a little change in reactions fluxes is made. Points that share the same shadow-price value are grouped in the same phenotypic phase.

#### Pareto analysis

This function allows to test trade-offs made by the cell between different objectives by comparing experimental values to FBA simulations. A list of potential cellular objectives (growth rate, minimising the sum of fluxes in the network, maximising ATP production …) and experimental flux measures are provided in an additional input file. The outputs are the cellular objectives for which the calculated Pareto surface is the closest to experimental values, meaning that they seem to participate to the cell’s trade-off.

This function is a generic implementation of the method proposed in [33] and represents, to our knowledge, the first implementation of this analysis in a flux analysis software.

#### Time-dependent FBA

This function is equivalent to the rFBA method described by Covert et al. [23]. The goal is to simulate the evolution of the system and environment (concentration of external metabolites) over time given an initial state, and monitoring both regulatory and metabolic networks states.

This method is not based on a steady-state analysis of the regulatory network, but on its dynamics. Here, a FBA is performed between each update of the qualitative network.

The algorithm takes as an input a metabolic network, a qualitative regulatory network, external metabolite concentrations (mmol/l), an initial cell density (g/l) and the identifier of the biomass reaction of the metabolic model. At each step, the values of the regulatory network are updated, translated into constraints for FBA. Then the cell density and external metabolite concentrations are updated by solving standard differential equations as detailed in [34].

This analysis allows simulating the production of metabolites over time, or the consumption of different nutrients by the cell (Fig. 3 panel c).

## Results

### Use case 1: steady-state analysis in different environmental conditions

Metabolic regulation via tuning of gene expression is paramount to understand cell behavior. This mechanism was first demonstrated in 1961 by Jacob and Monod on the *lac* operon which triggers a diauxic shift corresponding to a sequential consumption of two substrates when both are available [35]. Computation of FBA without considering the catabolite repression events fails in predicting this behaviour [36].

**Fig. 4:**
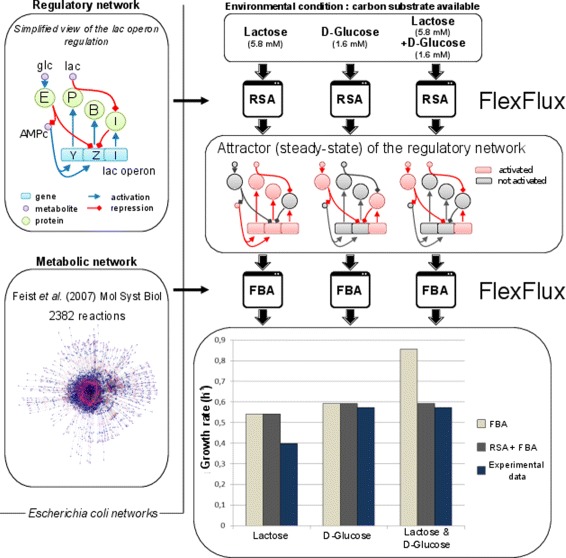
Simulations of *Escherichia coli* growing with different combinations of lactose and D-glucose. Simulations were performed using the genome-scale metabolic model of *E. coli* K-12 MG1655 [37] and the reconstructed regulatory network of the lac operon. Are shown here the initial concentrations of the two carbon sources, the calculated steady-state (attractor of size one) qualitative value for some of the components of the regulatory network, and the value of the objective function (growth rate). Lower graph legend : FBA: Simulation performed using FBA without considering the regulatory network; RSA + FBA: Simulation performed using RSA and FBA; experimental data: values extracted from [36]. Regulatory network legend: E: EIIA component of the D-glucose PTS transporter; P: lactose permease; B: Beta-galactosidase; I: lac I regulator

In order to illustrate FlexFlux’s capabilities in combining gene regulation and FBA, we performed simulation of the biomass production by *Escherichia coli* in environments composed of lactose and/or D-glucose using the reconstructed genome-scale metabolic model of *E. coli* [37] and a qualitative model of the *lac* operon [38, 39] that we translated into SBML-qual format (see Additional file 2). Initial substrate concentrations were extracted from [36] (Additional file 3) and we performed a RSA and a FBA with maximization of the biomass (Fig. 4).

In the three conditions, the initial values of the carbon sources available will generate different series of updates of the regulatory network and the calculation of three different attractors. All these attractors contain only one network state (Fig. 4). They correspond to two distinct cell phenotypes: utilisation of glucose and utilisation of lactose by the cell as a substrate. We compared these results with a FBA performed without using the regulatory network. In the latter case, the calculated objective function value does not correspond to the experimental data when both substrates are available.

The simulation results using RSA are consistent with the experimental data shown in [36] as well as the simulation performed using time-dependent FBA (rFBA) simulations [36]. However RSA does not require time-dependent simulations. It is able to quickly pinpoint the regulatory network and consequently the metabolic network steady-states by just changing input metabolite concentrations. In the case of time-dependent FBA, choosing the time step intervals for the simulation implies a trade-off between the risk of propagating an error due to the numerical solution in case of a long time step and the high computation time in case of a short time step.

This analysis shows that FlexFlux can easily find different metabolic behaviours according to environmental conditions. This is made possible by the steady-state analysis of the regulatory network provided alongside the metabolic network, and by the translation of qualitative values into continuous intervals allowing flux analyses.

In order to better evaluate the accuracy of RSA, we assessed whether RSA can obtain the same results than rFBA in most cases with a significantly lower number of calculations. Indeed, dynamic methods to regulate FBA can take into account a feedback of FBA results on regulatory rules but require many optimisations and updates of cell density and metabolite concentrations at each iteration. We used the data shown in [40] where authors compared rFBA results to experimental data for growth phenotypes of *E. coli* in 125 conditions and for 110 gene knock-outs. We found that in 93 % of the conditions tested (12797/13750), RSA was able to obtain the same growth phenotypes as rFBA and reaches an accuracy of 0.737 in predicting the experimental data (Additional file 4). The accuracy of rFBA to predict experimental data is 0.787.

Analysis of the discrepancy between false prediction of the RSA compared to rFBA indicates that RSA failed to predict the phenotype when a change in the extracellular metabolite concentration is required to activate assimilation pathway. This is expected considering that it is a steady-state method and not a dynamic one. In most of the cases with discrepancy, first acetate accumulation in the medium is required before activation of the glyoxylate cycle enzymes which are required to assimilate the main substrate. Adding the presence of this accumulated metabolites in the constraint file before running the RSA leads to obtain an identical prediction to rFBA. In this case, the prediction accuracy of RSA reaches the same level as rFBA.

This shows that RSA can be used as a replacement of rFBA in the majority of the conditions. RSA requires only one linear programming optimisation whereas rFBA may require hundreds. However, in few case, when the effect of metabolite concentrations is necessary for the simulation, a dynamic method is more adapted. This is what we implemented in FlexFlux in the Time-Dependent FBA function. Both methods (RSA and Time-Dependent FBA) support multi-state components and can constrain each reaction to multiple flux values depending on these states.

### Use case 2: multi-state logical modelling

To show an example where the multi-state capability of FlexFlux can be useful, we performed another analysis using the Jacob and Monod’s model showing the effect of glucose concentration on the catabolite repression of maltose and lactose in *E. coli*.

The catabolite repression is a well known regulation of substrate usage by microorganisms when “preferred” substrate is present in the medium. However, there is rising evidence that the catabolite repression is not fully operating at low substrate concentration, below the milimolare range. This could be relevant in many ecological niches. In addition, the strength of the repression, i.e the concentration of the preferred substrate in the medium under which the repression is fully operating, can greatly differ depending of the regulatory circuit of the second substrate usages. To illustrate the interest of multi-state modelling, we inferred the capacity of *E. coli* to use lactose and maltose for various concentrations of glucose. The regulatory network of the lac operon used in the paper was completed with the reconstruction of the regulation of the maltose operon [41], and then was converted in the SBML-qual format for computation (Additional file 2). The threshold of D-glucose concentration, 0.6 mM, under which the induction of the maltose operon starts was collected from [42] (Additional file 5). Then, we compared the capacity of the substrate usages simulated with the experimental data collected in [43], from cells grown on batch culture with high amount of D-glucose (22 mM) and cells grown on D-glucose limited (Additional file 6: Figure S1). Using multi-state modelling allows to simulate the capacity of the cells to use maltose but not lactose at low D-glucose concentration, i.e. below 0.6 mM but above 0 mM. This behaviour would not have been possible to simulate using Boolean modelling where D-glucose would harbour only two states.

### Use case 3: analysis of a large regulatory network

The challenge in genome scale analyses is that exploring the entire space of solution of a large scale network (over 100 degrees of liberty) remains infeasible. Hence if FBA and linear programming assure exploring boundaries of solution space of a genome scale metabolic network using an optimisation principle, an optimisation function can’t be applied in regulatory networks composed of feed-back loops. Indeed, none optimal state can’t be reached if a loop is involved within it. Thus, FlexFlux is designed to perform a random sampling of a significant proportion of the initial condition space to deal with large-scale regulatory networks.

**Fig. 5:**
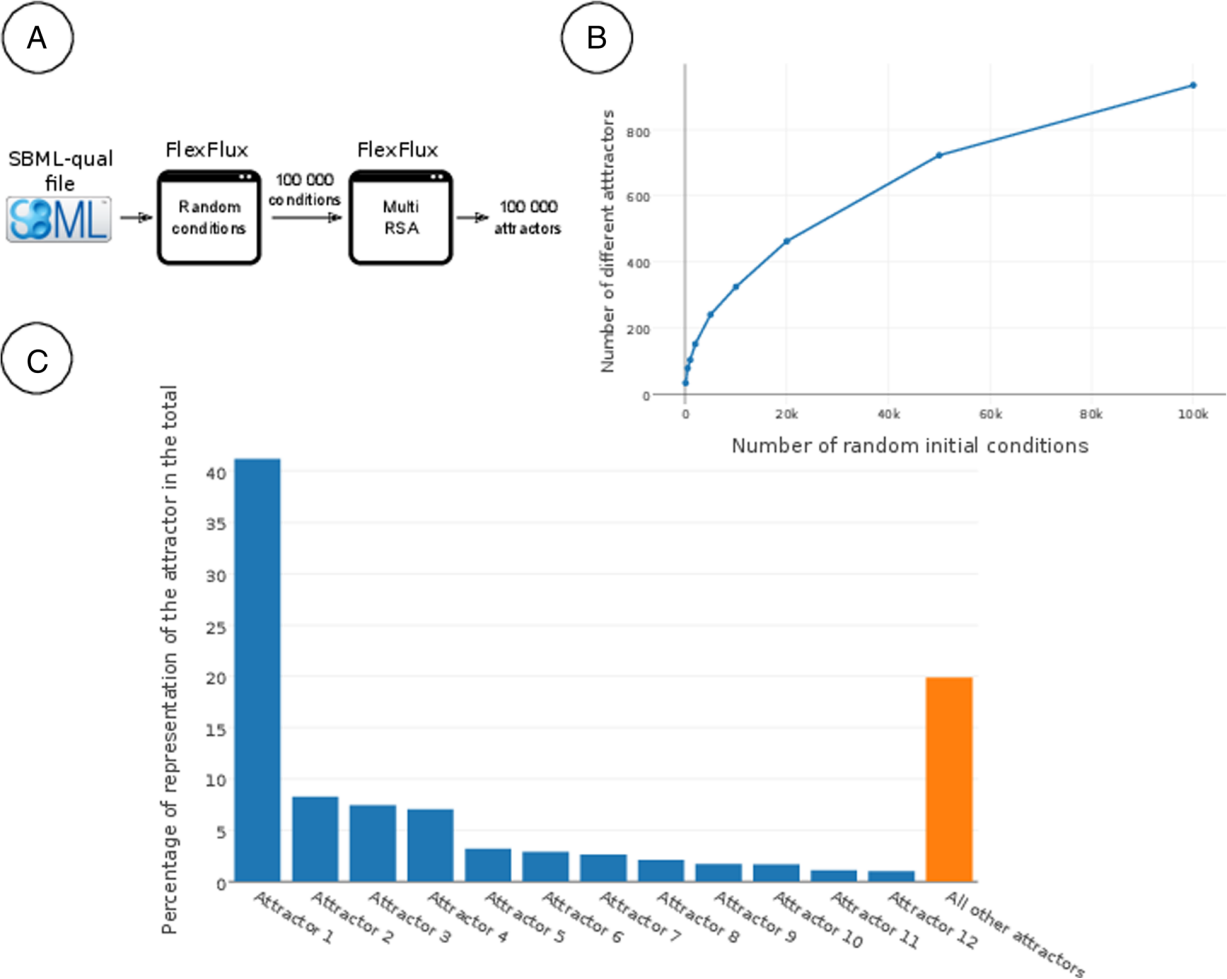
Results of a multi RSA performed on a large regulatory network. **a** Pipeline of FlexFlux functions to obtain 100.000 attractors from the initial regulatory network in the SBML-qual file format. **b** Evolution of the number of different found attractors in function of the number of initial conditions tested. **c** Percentage of representation of the attractors representing more than 1 % of the 100.000 attractors. The value in orange corresponds to the addition of all other 923 attractors which individually represent less than 1 % of the total

To test a large and publicly available regulatory network, we extracted a network from The Cell Collective [17] in the SBML-qual file format (see Additional file 7). This network corresponds to a generic fibroblast cell and was published in [44]. It contains 139 species and 260 FunctionTerms (which correspond to variables update rules). We performed this analysis by using a pipeline of two FlexFlux functions. We first generated 100.000 different random initial conditions for the regulatory network and then used them to run a multi RSA (Fig. 5). The output of the multi RSA is 100.000 regulatory network attractors. We then grouped the identical attractors together to count the proportion of each one (Fig. 5).

This analysis shows that FlexFlux can easily find steady-states that are dominant. Indeed, from the 100.000 initial conditions, FlexFlux found 935 different attractors, but the 12 most frequent of them represent more than 80 % of the total, with one of them representing more than 41 %. Sensitivity to the number of simulations was assessed by simulating various number of initial conditions. We obtained a logarithmic curve suggesting a correct sampling for 100.000 initial conditions (Fig. 5). This shows that, in the presence of a network for which it is not possible to test every possible initial condition (2^139^ initial conditions are possible for this network), FlexFlux is able to pinpoint the dominant states that the network can reach. These dominant states may then be used to constrain a metabolic network and perform flux balance analyses. We checked the accuracy of the RSA method on this Boolean network using BoolNet, and found identical results.

Also this result shows that FlexFlux can run a very high number of RSA in a limited time. We compared the computation time of steady-state analyses in different software in Additional file 1 (Table S2).

## Discussion

In order to realistically analyse metabolic networks, it is essential to consider them in interaction with regulatory networks. Also, one of the challenges in systems biology is taking charge of large-scale networks. FBA and logical modelling are classical methods used to perform simulations on large-scale metabolic and regulatory networks, respectively.

In this paper, we describe FlexFlux, a Java framework for integrating the analysis of these two networks. When provided, FlexFlux first calculates the steady-state of the regulatory network. This is used to define additional constraints that are applied to the metabolic model. FlexFlux is also able to translate qualitative variables from the regulatory network into quantitative variables used in FBA, thanks to a match list provided by the user. This possibility, for the first time implemented in a flux analysis software, can highly refine the modelling of the system. The multi-state qualitative models supported by FlexFlux allow subtle analyses with variables containing more than two possible states, in particular reaction fluxes. Finally, to our knowledge, FlexFlux is the only available tool that uses standard exchange file formats for both networks (SBML and SBML-qual), allowing full compatibility with other FBA or regulatory network analysis tools.

In order to determine the regulatory network steady-state, FlexFlux uses a synchronous update of the network state. As explained before, in the synchronous update case, a state completely determines the next one. This means that from a given initial network state, the same attractor will always be found. Moreover, the number of possible states for each component being finite, the number of possible network states is also finite and an attractor will always eventually be found. As described in [45] and [14], in the asynchronous update case, genes can be updated following different rules (time delay for each gene, random update …). This type of update is more suitable for studying the dynamics of a system. However, asynchronous update requires additional parameters and, in most cases, does not give better results than synchronous update in the search for attractors [45]. Also, synchronous update allows to perform simulations knowing the regulatory network structure alone. For this reason, we believe that synchronous update is a reasonable choice for identifying regulatory network steady-states in FlexFlux. However, one could be interested to use FlexFlux to study the dynamics of the system. Thus we implemented the possibility to use an asynchronous update based on time delays. This method is used in the time-dependent method of FlexFlux described above. This is a deterministic method, meaning that from the same inputs, the result will always be the same. FlexFlux does not contain methods based on random asynchronous updates that would be non-deterministic. Adding this functionality will be considered for a future improvement.

As detailed previously, new FBA constraints are set considering the attractor found in the regulatory network steady-state analysis. In the case of a cyclic attractor, each constraint is defined by the mean of the bound values corresponding to each component state in the attractor. We have chosen to use the mean of the states because we consider that it is more suitable to a broad range of studies when multiple cells are not synchronised. Indeed, when considering a whole population with multiple cells at different states, different states of the attractor will be encountered. Since the method is not able to evaluate the importance of the different states, they will all be considered as equally important for the steady-state of the cell. In consequence, the mean of the translated values simulates an average of the attractor states in the population. A different strategy could be to perform a FBA for each state of the attractor. This can still be done in FlexFlux by setting each state of the attractor as a constraint in separate FBA analyses.

Lastly, the fact that FlexFlux does not analyse both types of networks in the same optimisation process allows a higher flexibility in the possible regulatory logical rules. For example, negative auto-regulation, which is very common in biological systems [46], as well as feedback loops in general, cannot be included in a linear optimisation problem where all equations must be true at the same time. With the FlexFlux steady-state algorithm, feedback loops can be included in the regulatory network. Indeed, in the case of a negative auto-regulation, FlexFlux will reach a cyclic attractor of the regulatory network.

FlexFlux already offers 13 functions. All of them support a Regulatory Steady-state Analysis (RSA). FlexFlux code is open-source and thus can be used by other developers to create new functions that use the capabilities described in this paper. In addition, as we show in Additional file 1 (Tables S1 and S2), FlexFlux implementations of successive FBAs and steady-states analysis are among the fastest compared to other software. Indeed, 11 s are required for a FVA of the 2214 reactions within the *E. coli* model, iAF1260, and 12 s for 10,000 RSA with the Tcell model.

To facilitate the reader’s understanding of FlexFlux features compared to other applications and methods, we have compiled two comparison Tables.

Table 1 compares different FBA applications in their ability to integrate regulatory networks into FBA. It mainly shows that FlexFlux contains methods to regulate FBA both in a dynamic manner (time-dependent analysis, Fig. 3) and with a regulatory steady-state analysis (RSA) which is the part that is stressed in this article. CellNetAnalyser [5] supports different types of analyses for both regulatory networks and metabolic networks but fails in the integration of the two. Indeed, CellNetAnalyser can only load each network type and compute them separately into distinct dedicated modes. In FlexFlux, both network types are encoded separately as well but are integrated on the fly during simulation.

**Table 1:** FBA software comparison. Features comparison of applications discussed in this article focusing on their ability to integrate regulatory networks analyses into FBA

SBML support					Regulated FBA				
Software	Free	Metabolic	Regulatory	Graphical	Command line for	Dynamic	Steady state	ODEs	EFM
	dependencies	networks	networks	interface	batch processing				
FlexFlux	Yes	Yes	Yes	Yes	Yes	Yes (Time	Yes	No	No
						dependent FBA)	(RSA + FBA)		
Cell net analyser	No	Yes	Only for export	Yes	Yes	No	No	Yes	Yes
COBRA	No	Yes	No	No	Yes	Yes	No	No	No
						(Dynami-cRFBA)			
COBRApy	Yes	Yes	No	No	Yes	No	No	No	No
OptFlux	Yes	Yes	No	Yes	No	No	No	No	Yes
SurreyFBA	Yes	Yes	No	Yes	Yes	No	No	No	Yes
FASIMU	Yes	Yes	No	No	Yes	No	No	No	No
SBRT	Yes	Yes	No	Yes	Yes	No	No	No	Yes

ODEs Ordinary Differential Equations, EFM Elementary Flux Modes

Table 2 compares different methods dedicated to regulate FBA. They are mainly separated in two types: dynamic methods and steady-state methods. Most of them do not have an associated software which makes them less convenient to use.

**Table 2:** Comparison of methods to regulate FBA

Method	Associated software	Requires	Dynamic	Steady-state	Multistate	Regulatory	Network
		external data	analysis	analysis	support	loops support	inference
RSA + FBA	Yes (FlexFlux)	No	No	Yes	Yes	Yes	No
RFBA	Yes (COBRA and FlexFlux)	No	Yes	No	COBRA: No FlexFlux: Yes	Yes	No
SRFBA	No	No	No	Yes	No	No	No
iFBA	No	No	Yes	No	No	Yes	No
PROM	No	Yes	Yes	No	No	Yes	Yes

## Conclusions

FlexFlux is a free, open source Java software that joins two types of steady-state based analyses that are usually performed separately: FBA and qualitative multi-state simulations. FlexFlux is the first tool to support both SBML and SBML-qual standards file formats to describe metabolic and regulatory networks respectively. This support ensures compatibility with other FBA software and qualitative network analysis software. Regulatory network simulations are based on synchronous updates of the network state and the result can be translated into continuous intervals used as constraints in the FBA. This allows to easily constrain a flux analysis depending on regulatory network reprogramming. FlexFlux also contains different methods to analyse in detail regulatory and metabolic networks in interaction. The methods implemented in FlexFlux are efficient to deal with genome-scale networks.

FlexFlux is designed for researchers looking for an accessible tool capable of performing sophisticated analysis of the relations between metabolic and regulatory networks.

## Availability and requirements

**Project name:** FlexFlux.**Project home page:**http://lipm-bioinfo.toulouse.inra.fr/flexflux/index.html**Operating system(s):** Linux, Windows.**Programming language:** Java.**Other requirements:** Java 1.7 or higher, CPLEX orGLPK.**License:** GNU LGPL.**Any restriction to use by non-academics:** none.

## Additional files

Additional file 1
**Performance comparison of FlexFlux with other software.** (PDF 84 kb)

Additional file 2
**Reconstructed regulatory network of** ***E. coli*** **containing catabolite repression of lactose and maltose in the SBML-qual format.** (TXT 35 kb)

Additional file 3
**Constraint file used to run FlexFlux simulations of the lactose operon.** (TXT 484 bytes)

Additional file 4
**Comparison between rFBA and RSA on 125 conditions and 110 gene knock-outs of** ***E. coli*** **regulatory and metabolic networks.** (XLSX 43 kb)

Additional file 5
**Constraint file used to run FlexFlux simulations of the lactose and maltose operon.** (TXT 939 bytes)

Additional file 6
**Results of the analysis of the lactose and maltose operon of** ***E. coli*** **.** (PDF 132 kb)

Additional file 7
**Fibroblast regulatory network [** 44 **] in the SBML format.** (TXT 505 kb)

## Abbreviations

ATP: Adenosine triphosphate
FBA: Flux balance analysis
FVA: Flux variability analysis
GLPK: GNU linear programming kit
GPR: Gene-protein-reaction associations
KO: Knockout
MILP: Mixed integer linear programming
ODEs: Ordinary differential equations
PROM: Probabilistic regulation of metabolism
rFBA: Regulatory FBA
RSA: Regulatory steady-state analysis
SBML: Systems biology markup language
SBML-qual: SBML qualitative models package (“qual”) extension
SR-FBA: Steady-state rFBA
XML: Extended markup language
