# Supplementary material for: FlexFlux: combining metabolic flux and regulatory network analyses
Source: BMC Syst Biol. 2015 Dec 15;9:93. doi: 10.1186/s12918-015-0238-z (PMC4678642; doi:10.1186/s12918-015-0238-z)
Supplement: Additional file 1 — Performance comparison of FlexFlux with other software. (PDF 84 kb) [file 12918_2015_238_MOESM1_ESM.pdf]

Additional file 1

FlexFlux: combining metabolic flux and regulatory network analyses

Lucas Marmiesse; Remi Peyraud; Ludovic Cottret

**Software**	FVA time (s)
FlexFlux	11
COBRApy	7
CellNetAnalyser	810
SurreyFBA	440
OptFlux	700

Supplementary table 1: Comparison of computation time of FVA in different software. Tests were performed on a Flux variability analysis (FVA) of all 2214 reactions of a metabolic model of *E.coli* : Ec iAF1260. Calculations were made using a computer computer with one processor (Intel ® Core ™ i5-4590).

**Software**	steady-state analysis time (s)
FlexFlux	12
BoolNet	60
CellNetAnalyser	80

Supplementary table 2: Comparison of computation time of steady-state analysis in different software. Tests were performed on 10000 consecutive steady-state analyses of a regulatory network provided in the CellNetAnalyser software (Tcell) which contains 95 components. Calculations were made using a computer computer with one processor (Intel ® Core ™ i5-4590).
