# Supplementary material for: FlexFlux: combining metabolic flux and regulatory network analyses
Source: BMC Syst Biol. 2015 Dec 15;9:93. doi: 10.1186/s12918-015-0238-z (PMC4678642; doi:10.1186/s12918-015-0238-z)
Supplement: Additional file 6 — Results of the analysis of the lactose and maltose operon of E. coli . (PDF 132 kb) [file 12918_2015_238_MOESM6_ESM.pdf]

**Additional file 6**

FlexFlux: combining metabolic flux and regulatory network analyses

Lucas Marmiesse; Remi Peyraud; Ludovic Cottret

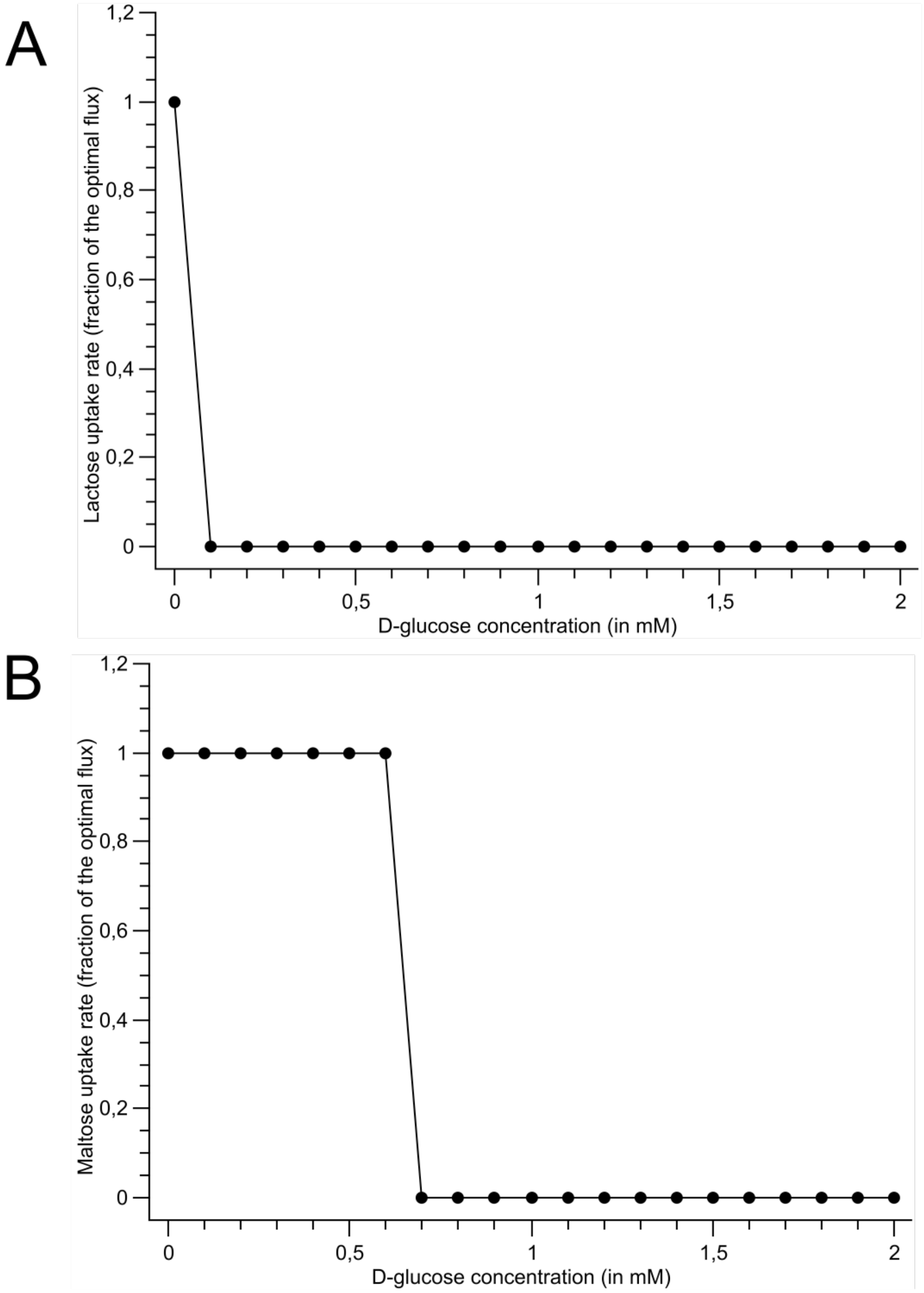


C

Respiration rate [ Δ $Ext_{460}$ $OD_{546}^{-1}$ $h^{-1}$]

| Substrate | Glucose limited chemostat | Batch-culture (22 mM D-glucose) |
|---|---|---|
| Lactose | 0.0 | 0.0 |
| Matlose | ~0.6 | 0.0 |

Data from Ihssen and Egli (2005) Environmental Microbiology

**Supplementary Figure 1.** Multi-state logical modelling of catabolite repression. Simulation of the *Escherichia coli* lactose uptake rate **(A)** and maltose uptake rate **(B)** depending of the D-glucose concentration in the medium. Simulations were conducted using the genome-scale of *Escherichia coli* (Feist *et al.*, 2007) and the reconstructed regulatory network describing the regulation of the lactose and the maltose operons. The panel **C** displays the

experimental data published by Ihssen and Egli in 2005 in Environmental Microbiology which demonstrate the maltose usage capacity of the *E. coli* cells grown under limited D-glucose concentration, whereas the lactose usage capacity is still repressed.
